# Preclinical safety profile of a liver-localized mitochondrial uncoupler: OPC-163493

**DOI:** 10.17179/excli2021-4414

**Published:** 2022-01-11

**Authors:** Yuki Inoue, Junichi Kino, Nobuya Ishiharada, Makoto Sato, Suguru Hatanaka, Hiroyuki Yokoi, Takahiro Shimada, Seiji Sato, Takashi Okamoto, Naohide Kanemoto

**Affiliations:** 1 Department of Drug Safety Research, Nonclinical Research Center, Tokushima Research Institute, Otsuka Pharmaceutical Co., Ltd., Tokushima, Japan; 2 Product Strategy Team 1, Product Strategy & Intelligence Office, Regulatory Affairs Department, Otsuka Pharmaceutical Co., Ltd., Tokyo, Japan; 3 Department of Investigative Toxicology, Nonclinical Research Center, Tokushima Research Institute, Otsuka Pharmaceutical Co., Ltd., Tokushima, Japan; 4 Department of Drug Metabolism and Pharmacokinetics, Nonclinical Research Center, Tokushima Research Institute, Otsuka Pharmaceutical Co., Ltd., Tokushima, Japan; 5 Medicinal Chemistry Research Laboratories, New Drug Research Division, Otsuka Pharmaceutical Co., Ltd., Tokushima, Japan; 6 Department of Lead Discovery Research, New Drug Research Division, Otsuka Pharmaceutical Co., Ltd., Tokushima, Japan

**Keywords:** OPC-163493, mitochondrial uncoupler (mUncoupler), liver toxicity, blood vessels toxicity, kidney toxicity

## Abstract

Mitochondrial uncouplers (mUncouplers) are known to exhibit a variety of toxic effects in animals. Here we report a safety profile of an mUncoupler, OPC-163493, recently synthesized at Otsuka Pharmaceutical Co, Ltd, and its development as a therapeutic agent for treating diabetes. To understand the acute and subchronic toxicity of OPC-163493, single and repeated oral dose studies in rats, dogs, and monkeys were performed. In the rat studies, rigor mortis and increased body temperatures were observed in the high dose group. Focal necrosis, fatty change, and granular eosinophilic cytoplasm of the hepatocytes were also observed in the high dose group. In the dog studies, gastrointestinal manifestations were observed with decreased body weight and decreased food consumption in the high dose group. Necrotizing arteritis was observed in multiple organs as well as meningitis with hemorrhage in the brain. In the monkey studies, vomiting, decreased food consumption, and decreased locomotor activity were observed in the high dose group. Degeneration of the proximal convoluted tubules and the straight tubular epithelium, regeneration of the proximal tubular epithelium, and degeneration of the collecting tubular epithelium were observed. The target organs of OPC-163493 were liver, blood vessels, and kidney in rats, dogs, and monkeys, respectively. In rats, dogs, and monkeys, safety ratios were 100:1, 13:1, and 20:1, respectively, in terms of total exposure (AUC_24h_). These safety ratios showed clear separation between exposure to OPC-163493 in animals at NOAEL and the exposure at the effective dose in ZDF rats. This information should contribute to the drug development of new and effective mUncoupler candidates.

## Introduction

OPC-163493, which has been developed for the treatment of diabetes, is a liver-localized/targeted mUncoupler (Kanemoto et al., 2019[12]; Okamoto et al., 2021[16]). 2,4-Dinitrophenol (DNP), a well-known drug that induces mitochondrial uncoupling, was used in diet pills in the 1930s. The use of DNP was banned by the United States government because of the occurrence of harmful effects including fatal hyperthermia and cataracts (Colman, 2007[2]; Horner, 1942[8]). On the other hand, the toxicological profile for DNP in animals has been reported in detail by the U.S. Department of Health and Human Services (Satcher, 1995[22]). Acute DNP intoxication has been well characterized in rats and dogs and symptoms include high fever, high heart rate and high respiratory rate. These changes were produced by an increase in metabolic rate. However, no clear features of subacute intoxication with DNP have been observed in animals. Cataracts, which was one of the reasons DNP was withdrawn from medical use, developed in a small percentage of patients at therapeutic doses of DNP (Horner et al., 1935[9]). However, animal studies have not been able to induce cataracts as observed in humans exposed to DNP. Due to the historical background of the severe toxicity of mUncoupler agents in humans and the toxic effects on sensory organs (cataracts) due to clinical exposure, mUncoupler agents are now widely perceived as toxic rather than therapeutic agents. However, there are species differences in the toxicity of mUncoupler agents, and no obvious serious toxicity has been observed in long-term toxicity studies (Satcher, 1995[22]). Nowadays there is a reviving interest in mUncoupling as a therapeutic strategy (Cunha et al., 2011[3]; Jastroch et al., 2013[10]) and several attempts (Fu et al., 2013[4]; Kanemoto et al., 2019[12]; Kenwood et al., 2013[14]; Okamoto et al., 2021[16]; Perry et al., 2013[20], 2015[21]; Tao et al, 2014[26]) to seek safe chemical mUncouplers have been conducted due to their energy consuming benefit. Among those attempts, liver-targeted mUncoupling proposed by Perry et al. is a particularly promising means of efficacious and safe treatment for diabetes and hepatic steatosis. However, both of two mUncouplers proposed by them are derivatives of DNP: one is a prodrug version (Perry et al., 2013[20]) and the other is a controlled-release formulation of DNP (Perry et al., 2015[21]). On the other hand, OPC-163493 is a completely new molecular entity developed as a liver-localized mUncoupler (Kanemoto et al., 2019[12]; Okamoto et al., 2021[16]). Therefore, it is thought that new therapeutic benefits may be generated by clinical use in humans and appropriate drug handling. 

## Materials and Methods

### Physical and chemical properties

Information regarding the physical and chemical properties of OPC-163493 can be found in Table 1(Tab. 1). OPC-163493 was obtained from the Saga Factory of Otsuka Pharmaceutical Co, Ltd. The test article was stored at room temperature and protected from light.

### Preparation of the test article for rat and monkey studies

The dosing suspensions were prepared in 5 % gum arabic solution (gum arabic; Sankyo Foods Industry Corp, water for injection; Otsuka Pharmaceutical Factory, Inc.) to make appropriate suspensions. The dosing solutions were prepared at least once weekly and stored in a cool place (1 °C to 10 °C) under protection from light. The concentrations and homogeneity of the test article in the dosing formulations were confirmed at the test facilities. The test article was confirmed to be stable in the vehicle at the concentrations of 0.04, 2, and 200 mg/mL for 1 day at room temperature under protection from light following storage for 8 days at temperatures not exceeding 10 °C under protection from light.

### Preparation of the test article for dog studies

The appropriate quantity was calculated based on the most recent body weight measured, and the test article was placed in 1 capsule (1/4-oz gelatin capsule, Torpac Inc.) for the test article-treated groups for daily dosing. The test article in the gelatin capsule was prepared on appropriate days and stored at room temperature (10 °C to 30 °C) and under protection from light until dosing.

### Animal husbandry

All studies were conducted in compliance with the Guidelines for Animal Care and Use in Otsuka Pharmaceutical Co, Ltd. or contract vendors, and were approved by the Institutional Animal Care and Use Committee of the testing facilities.

Sprague-Dawley rats (Crl:CD[SD]) were used for the safety pharmacology, genotoxicity, and general toxicity studies. On the day of administration, the rats were 6 or 7 weeks old, and body weights ranged from 193 to 330 g for male and 145 to 221 g for females in the safety pharmacology and the general toxicity studies. For the genotoxicity studies, the rats were 8, 9, or 10 weeks old, and body weights ranged from 288 to 406 g for males on the day of administration. All rats were purchased from the Charles River Japan, Inc., and were acclimated for at least 7 days prior to first dosing. The rats were individually housed in stainless-steel cages in a room under a 12-hour light-dark cycle, ventilated with an air-exchange rate of 6 to 20 times per hour, and maintained at 19 °C to 25 °C with a relative humidity of 35 % to 75 %.

Seven- to 8-month-old male and female beagle dogs with body weights ranging from 7.0 to 10.4 kg for males and 6.5 to 9.1 kg for females, bred by Covance Research Products Inc., were purchased from Japan Laboratory Animals Inc. All dogs were acclimated to laboratory conditions for 6 weeks or more prior to dosing. The dogs were vaccinated twice with Duramune®MX8 (Fort Dodge Animal Health, USA) and bacteriological and protozoan parasite tests were routinely conducted to examine the microbiological status of each dog. The dogs were individually housed in stainless-steel cages in a room under a 12-hour light-dark cycle. The room was ventilated with an air-exchange rate of 13 to 17 times per hour, and maintained at 21 °C to 25°C. The relative humidity was 50 % to 70 %. Transient increases in the humidity exceeding the upper limit and decreases in the air-exchange rate below the lower limit occurred in the housing room.

Three- to 6-year-old male and female cynomolgus monkeys (*Macaca fascicularis*) and with body weights ranging from 2.9 to 5.3 kg for males and 2.5 to 3.6 kg for females, were purchased from Gaoyao Kangda Laboratory Animals Science & Technology Co., Ltd., Guangdong, China. All monkeys were acclimated to laboratory conditions for 5 weeks or more prior to dosing. The monkeys were individually housed in stainless-steel cages in a room under a 12-hour light-dark cycle. The room was ventilated with an air-exchange rate of 10 to 30 times per hour, and maintained at 23 °C to 29 °C. The relative humidity was 35 % to 75 %. 

Diets were procured from commercial vendors. Animals were provided Otsuka's in-house tap water or filtered and irradiated water. No contaminants were present in the diet or water at levels which might have affected the outcomes of the studies.

### Histopathology

The following organs or tissues of all animals were fixed in 10 % neutral buffered formalin: liver, gallbladder (dog and monkey), kidneys, ureters, thymus, mandibular lymph nodes, mesenteric lymph nodes, spleen, heart, aorta, lungs with bronchi, trachea, larynx, esophagus, submaxillary glands, sublingual gland, parotid glands, tongue, stomach, duodenum, jejunum, Peyer's patch, ileum, cecum, colon, rectum, pancreas, urinary bladder, seminal vesicles, prostate, ovaries, oviducts, uterus, uterine cervix, vagina, pituitary gland, thyroid glands, parathyroid glands, adrenal glands, skin, mammary glands, skeletal muscle, brain, spinal cord, optic nerve, harderian glands (rat), sciatic nerve, sternum with bone marrow, femur with bone marrow and stifle joint. The eyeballs were fixed in Davidson's solution, and the testes and epididymides were fixed in FSA (formalin-sucrose-acetic acid) solution. The sternums, femurs, vertebrae and stifle joints were decalcified after fixation. According to the standard methods, the tissues were embedded in paraffin. For all animals of the control and high-dose groups, the paraffin block tissues were thinly sectioned and stained with hematoxylin and eosin (H.E.), and light microscopic examination was conducted. The kidneys, testes and epididymides were further stained with periodic acid-Schiff (PAS).

### Electron microscopy in liver and kidney after 4-week toxicity studies in rats and dogs

The samples of the liver and kidney (right cortex) were obtained from 2 males and 2 females of the control and high dose groups after the dosing and recovery periods. The tissue samples obtained were pre-fixed in a mixture of 2.5 % glutaraldehyde and 2 % paraformaldehyde, post-fixed in 1 % osmium tetroxide, stained en bloc with uranyl acetate, dehydrated with a graded series of acetone, and embedded in epoxy resin. Depending on the histopathological findings, the livers (central zone) of the animals of the control and high dose groups were subjected to the transmission electron microscopy. In addition, the liver (intermediate zone) of the animal of the high dose group was subjected to transmission electron microscopy. The specimens embedded in epoxy resin were sectioned ultra-thinly using an ultramicrotome, stained with lead citrate, and observed with a JEM-1400Plus transmission electron microscope (JEOL, Japan). In the animals after completion of the recovery period, the transmission electron microscopy was not conducted in the liver because there was no test article-related abnormality in the liver at the end of the recovery period. For the kidney samples obtained at the end of the dosing and recovery periods, the transmission electron microscopy was not conducted because there was no test article-related abnormality in the kidney.

### Statistical analysis

The following data obtained during the dosing period were analyzed by one-way analysis of variance for homogeneity in rats, dogs and monkeys; body weight, body weight gain, food consumption, body temperature and organ weights as well as the results of hematology, blood biochemistry, electrocardiography except for the cardiac rhythms, electroretinography, and urinalysis (except for the semiquantitative paper test). When significant (p < 0.05) difference was found in one-way analysis of variance, Dunnett's test was used for comparisons between the vehicle control and treated groups. Urine pH was analyzed by the Dunnett's test using rank transformation, when significant (p < 0.05) difference was found in the Kruskal-Wallis rank test for homogeneity of variance. For the analysis of the other semiquantitative urinalysis data, exact rank-sum test for 2 × k table was used. A paired t-test against the data recorded before the dosing period was performed for the results of hematology in dogs and monkeys, blood biochemistry and urinalysis (quantitative), and the data recorded before the dosing on Day 1 was also performed for the results of electrocardiography except for the cardiac rhythms, body temperature, and electroretinography. Regarding the data from the recovery period in rats, the t-test with F-test for homogeneity of variance was used for the inter-group comparisons of the body weight, body weight gain, food consumption, and organ weights as well as hematology, blood biochemistry, and urinalysis (except for the semiquantitative paper test). Wilcoxon rank sum test was used for the inter-group comparisons of urine pH, and inter-group comparisons of semiquantitative urinalysis data, other than urine pH, were performed using exact ranksum test for 2 x k table. For the recorded data in the 4-week recovery period, statistical analysis was not conducted in dogs and monkeys. The significance tests were performed in two-tailed analyses and statistical significance was presented either at the 5 % or 1 % level.

### Toxicokinetics

Approximately 0.5 mL of blood was collected from the jugular vein into a heparinized syringe under unanesthetized condition from all animals of the TK group. Blood samples were collected at 1, 2, 4, 8 and 24 hours post-dose on Days 1 and 14. The plasma concentrations of OPC-163493 and M29501 (major metabolite: Acyl Glucuronides; Supplementary Table 1) were determined using high-performance liquid chromatographic-electrospray ionization tandem mass spectrometry.

### Tissue concentrations in liver and kidney after 4-week toxicity studies in rats, dogs, and monkeys

Liver and kidney samples were obtained from 4-week oral toxicity studies in rats (300 mg/kg), dogs (100 mg/kg), and monkeys (50 mg/kg). The quantitative assay for OPC-163493 and M29501 in the liver and kidney homogenates used the high-performance liquid chromatographic-electrospray ionization tandem mass spectrometry (LC-MS/MS) method.

### Experimental designs

Oral gavage was selected due to the intended clinical route of OPC-163493. Dose settings, duration periods and animal numbers were in accordance with OECD and ICH guidelines (Supplementary Table 2). Observations, measurements, and examinations for toxicity studies are shown in Supplementary Table 3.

## Results

### Single dose toxicity in rats (Non-GLP)

Death occurred in 1 female at 1000 mg/kg and in all males and females at 2000 mg/kg. The approximate lethal dose was estimated to be between 1000 and 2000 mg/kg for male rats and between 300 and 1000 mg/kg for female rats. The results of observations, measurements, and examinations are shown in Supplementary Table 4.

### Preliminary 2-week repeated dose toxicity study in rats (Non-GLP)

**Figure 1:**
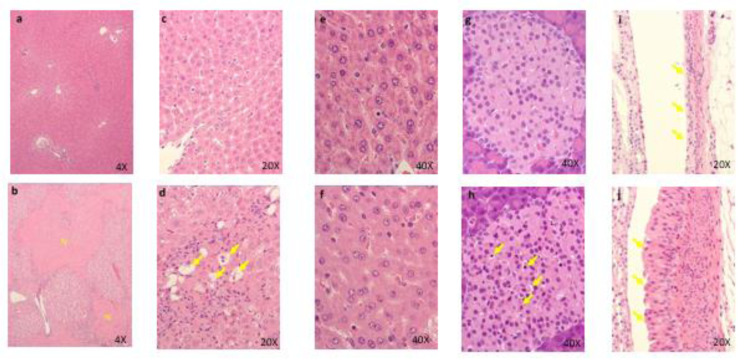
Histopathological findings in liver. control (a)(c)(e), 600 mg/kg (b)(d)(f); a, No abnormalities detected. b, Focal necrosis of hepatocytes in the central zone of lobules( N ) are seen. c, No abnormalities detected. d, Many balloon-like hepatocytes (arrows) are seen. No abnormalities detected. e, No abnormalities detected. f, Diffuse granular eosinophilic cytoplasm of hepatocytes are seen. No abnormalities detected. Histopathological findings in pancreas. control (g), 600 mg/kg (h), g, No abnormalities detected. No abnormalities detected. h, Several necrotic islet cells (arrows) are seen. No abnormalities detected. Histopathological findings in kidney. control (i), 600 mg/kg (j), i, No abnormalities detected. No abnormalities detected. j, Hypertrophy of transitional cells are seen. Arrows: transitional epithelium

Three males and 3 females died or were sacrificed moribund at 600 mg/kg/day. Hypoactivity, salivation, and flaccidity and dilatation of the scrotum, as well as body weight loss with decrease in food consumption, were noted in the early stage of the dosing period. In these animals, the histopathology showed focal necrosis of hepatocytes, necrosis of hepatocytes in the central zone of lobules, necrosis of subcapsular hepatocytes, balloon-like hepatocytes, hypertrophy of hepatocytes, and granular eosinophilic cytoplasm of hepatocytes in the liver, hypertrophy of transitional cells in the renal pelvis, ureter, and urinary bladder, increase of extramedullary hematopoiesis in the spleen, and necrosis of the islet cells in the pancreas (Figure 1(Fig. 1)). The plasma concentrations of OPC-163493 were elevated in a dose-related manner in both sexes, and no remarkable gender differences were observed. The results of observations, measurements, and examinations are shown in Supplementary Table 5.

### Four-week repeated dose toxicity study with 4-week recovery test in rats (GLP)

**Figure 2:**
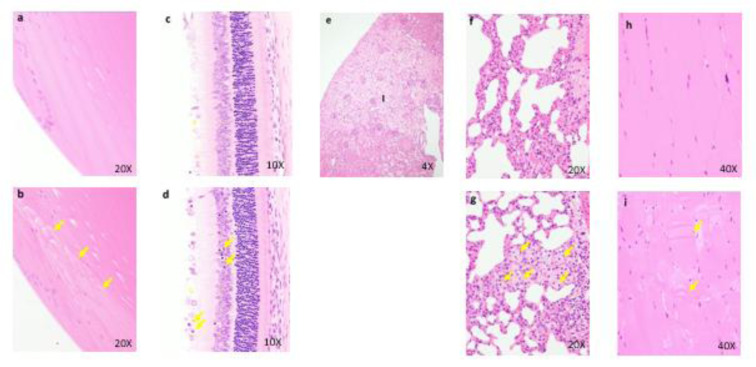
Histopathological findings in lens. control (a), 300 mg/kg (b), a, No abnormalities detected. b, Degeneration of lens fibers (arrows) are seen. Histopathological findings in retina. control (c), 300 mg/kg (d), c, No abnormalities detected. d, Apoptotic cells (arrows) are seen. Histopathological findings in kidney (e), 300 mg/kg, The infarction (I) is seen. Necrosis and inflammatory cells infiltration in the lesions. Histopathological findings in lung. control (f), 300 mg/kg (g), f, No abnormalities detected. g, Many foamy cells ( arrows ) are seen. Histopathological findings in skeletal muscle. control (h), 300 mg/kg (i), h, No abnormalities detected. i, Degeneration/necrosis of muscle fibers (arrows) are seen.

**Figure 3:**
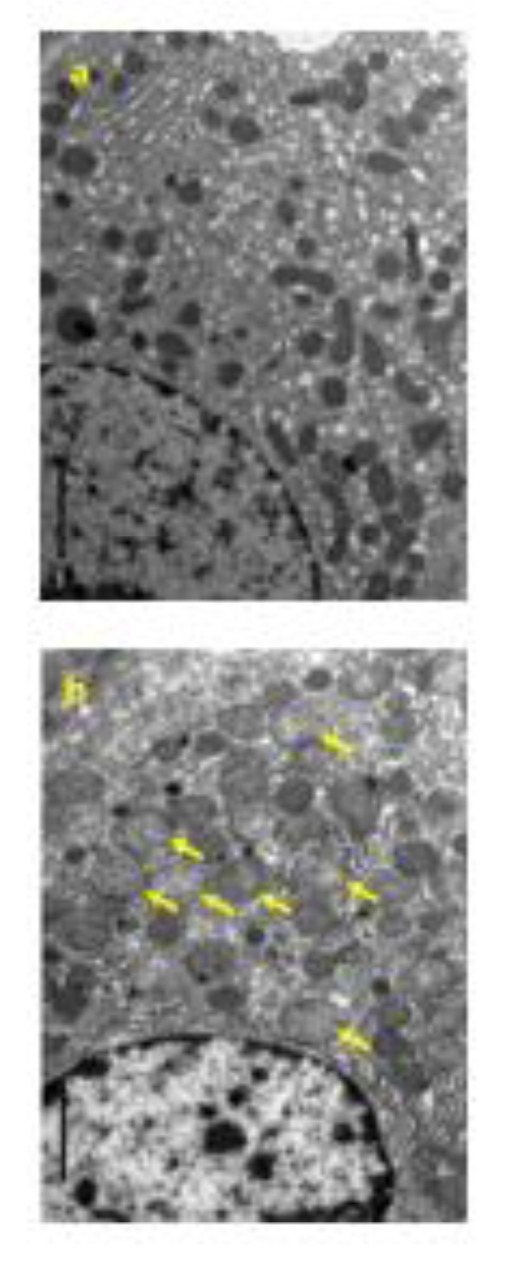
Electron micrograph of the hepatocyte in the liver. control (a), 300 mg/kg (b), a, Normal shaped mitochondria are seen. b, Enlargement of mitochondria (arrows) are seen. (Original magnification x 5,000)

Four females died at 300 mg/kg/day. Emaciation, stains around the nose, stains in the periproct, loose stool, reddish urine, and salivation, as well as body weight loss with decrease in food consumption, were noted. In these animals, the histopathology showed hyperplasia of the transitional cells in the renal pelvis and urinary bladder, necrosis of the islet cells in the pancreas, and degeneration/necrosis of the muscle fibers. New histopathological findings that were not observed in the 2-week study, degeneration of lens fibers, apoptotic cells in retina, the infarction in kidney, foamy cells in lung and degeneration/necrosis of muscle fibers in skeletal muscle observed in the 300 mg/kg group (Figure 2(Fig. 2)). Enlargement of mitochondria was observed in the hepatocyte (Figure 3(Fig. 3)). The changes noted during the dosing period in the 300 mg/kg group were no longer evident during the recovery period. The no observed adverse effect level (NOAEL) was estimated to be 30 mg/kg/day for males and 100 mg/kg/day for females. At the NOAEL, the plasma Cmax and AUC24h of OPC-163493 at Week 4 were respectively 12.03 µg/mL and 99.90 µg·h/mL in males and 49.35 µg/mL and 397.6 µg·h/mL in females. The results of observations, measurements, and examinations are shown in Table 2(Tab. 2).

### Thirteen-week repeated dose toxicity study in rats (GLP)

No deaths occurred at any dose. Hypertrophy of the hepatocytes was observed in the 30 and 100 mg/kg groups. Slightly increased liver weight was observed in the 100 mg/kg group. In addition, slightly increased TBI and CHO were observed in the 100 mg/kg group. These changes were considered to be of no toxicological significance since no damage or degenerative changes of the liver cells were observed in the group. The NOAEL was estimated to be 100 mg/kg/day for both males and females. At the NOAEL, the plasma Cmax and AUC24h of OPC-163493 at Week 13 were respectively 36.16 µg/mL and 459.1 µg·h/mL in males and 56.16 µg/mL and 477.0 µg·h/mL in females. The results of observations, measurements, and examinations are shown in Supplementary Table 6.

### Single dose toxicity in dogs (Non-GLP)

No deaths occurred at any dose. Vomiting, diarrhea, loose stool, and low food consumption were transiently observed in both sexes at 300 and 600 mg/kg. The results are shown in Supplementary Table 7.

### Preliminary 2-week repeated dose toxicity study in dogs (Non-GLP)

**Figure 4:**
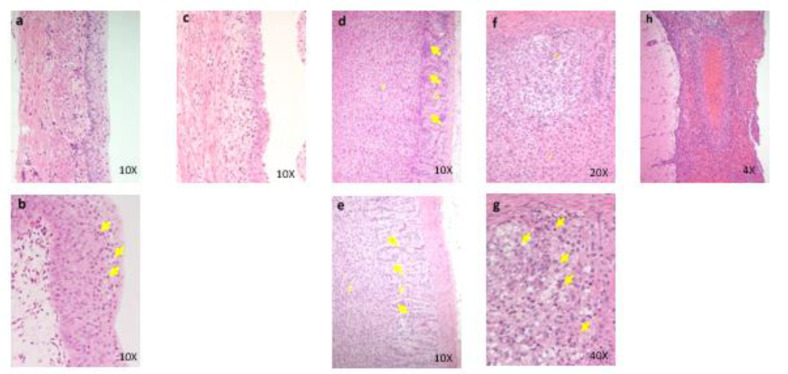
Histopathological findings in renal pelvis in kidney. control (a), 100 mg/kg (b), 600 mg/kg (c), a, No abnormalities detected. b, Many necrotic cells (arrows) are seen in the mucosa. c, Thinning of the mucosal epithelium. Histopathological findings in adrenal. control (d), 600 mg/kg (e)(f)(g), d, No abnormalities detected, e, Hypertrophy of the cortical cells at glomerulosa/fasciculate boundary (arrows) are seen. f, g, Necrosis and degeneration of the glomerulosa cells are seen. Degenerative cells show vacuolated cytoplasma. Many necrotic cells (arrows) are also seen in the same lesion. g: zona glomerulosa, f: zona fasciculate, arrows: glomerulosa/ fasciculate boundary. Histopathological findings in brain. 600 mg/kg (h), h, Inflammatory cells infiltration and hemorrhage are seen in the meninges.

No deaths occurred at any dose. However, marked decreases in food consumption and body weight with deterioration of general condition were observed in one female in the 600 mg/kg group in the last 2 days of the administration period. Vomiting, diarrhea, mucous stool, loose stool, and/or stool with red materials were observed in the treated animals. Salivation was observed in the 600 mg/kg group. Decreased body weight was observed in the 100 and 600 mg/kg groups, and low food consumption was observed in the 600 mg/kg group. For the hematology and blood biochemistry, the following changes were observed in the 600 mg/kg group: increased WBC, Neut, BUN, ALP, CHO, PL, and Bil and decreased CRE, BUN, and Glu. Abnormal contents in the gallbladder were observed in the 600 mg/kg group. For the histopathology, the following changes were observed in the 600 mg/kg group: thinning and increase of mitotic figures in the mucosal epithelium in the renal pelvis, hyperplasia of the mucosal epithelium covering the renal papilla, pyelitis, hyperplasia and thinning of the mucosal epithelium of the ureter and urinary bladder, increase of mitotic figures in the mucosal epithelium of the ureter, hypertrophy of the cortical cells at the glomerulosa/fasciculata boundary, necrosis and degeneration of the zona glomerulosa cells in the adrenal glands, focal atrophies of the parotid, submaxillary, and zygomatic glands, lobular atrophy of the sublingual gland, nesidioblastosis in the pancreas, meningitis with hemorrhage in the brain, and meningitis of the spinal cord (Figure 4(Fig. 4)). Necrosis, thinning, and hyperplasia of the mucosal epithelium in the renal pelvis was observed in the 100 mg/kg group. Meningitis with hemorrhage in the brain was observed in the 100 mg/kg group. The results of observations, measurements, and examinations are shown in Supplementary Table 8.

### Four-week repeated dose toxicity study with 4-week recovery test in dogs (GLP)

**Figure 5:**
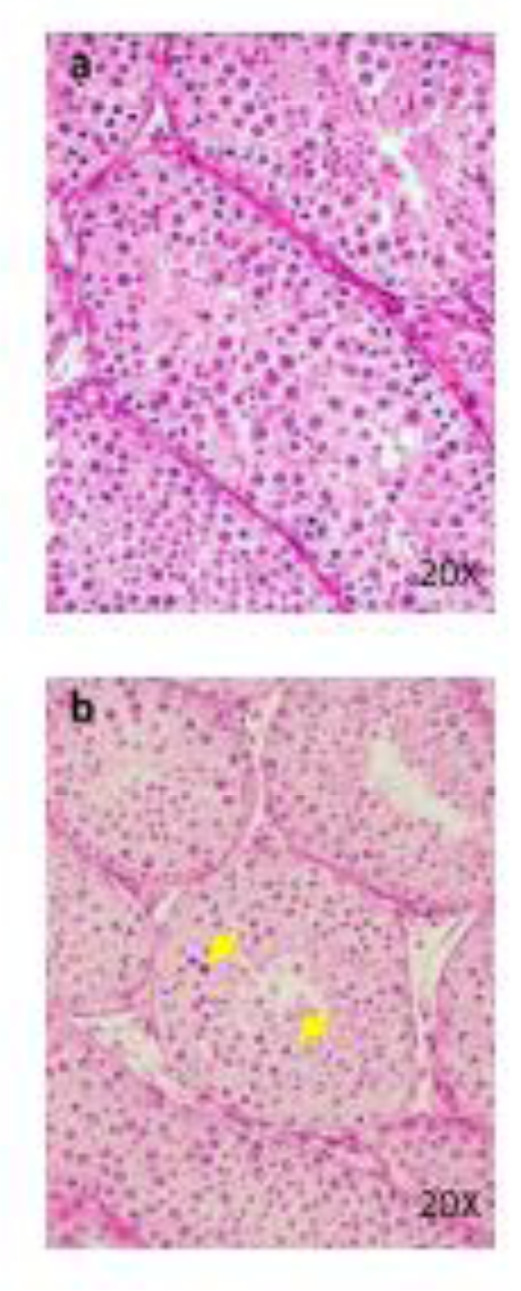
Histopathological findings in testis. Conrol (a), 100 mg/kg (b), a, No abnormalities detected. b, Degeneration/ necrosis of germ cells (arrows) are seen.

No deaths occurred during either the dosing or recovery period. However, decreases in food consumption and body weight were observed in 1 male in the 100 mg/kg group at Week 2 and thereafter. For the other treated animals, vomiting, diarrhea, and loose stool were observed in the 100 mg/kg group. Decreased body weight was observed in the 100 mg/kg group. Increased Cl and Neut were observed in the 100 mg/kg group. Positive reactions for occult blood and RBC in the sediment were observed in the 100 mg/kg group. Small amounts of abnormal contents in the gallbladder were observed in the 30 and 100 mg/kg groups. In addition to the systematic arteritis observed in the 2-week study, enlargement of the medial iliac lymph nodes, yellowish brown pigment deposition in the hepatocytes and necrosis of the mucosal epithelium in the renal pelvis were observed in the 100 mg/kg group. New histopathological findings that were not observed in the 2-week study, degeneration/necrosis of germ cells in the testis was noted in the 100 mg/kg group (Figure 5(Fig. 5)). The changes noted during the dosing period were no longer evident during the recovery period in the clinical observations, body weights, food consumption, hematology, blood biochemistry, necropsy, or organ weights. The plasma concentrations of OPC-163493 were elevated in a dose-related manner in both sexes, and no remarkable gender differences were observed. The NOAEL was estimated to be 10 mg/kg/day for both males and females. At the NOAEL, the plasma C_max_ and AUC_24h_ of OPC-163493 at Week 4 were respectively 5.193 µg/mL and 31.89 µg·h/mL in males and 7.614 µg/mL and 54.24 µg·h/mL in females. The results of observations, measurements, and examinations are shown in Table 3(Tab. 3). 

### Thirteen-week repeated dose toxicity study in dogs (GLP)

**Figure 6:**
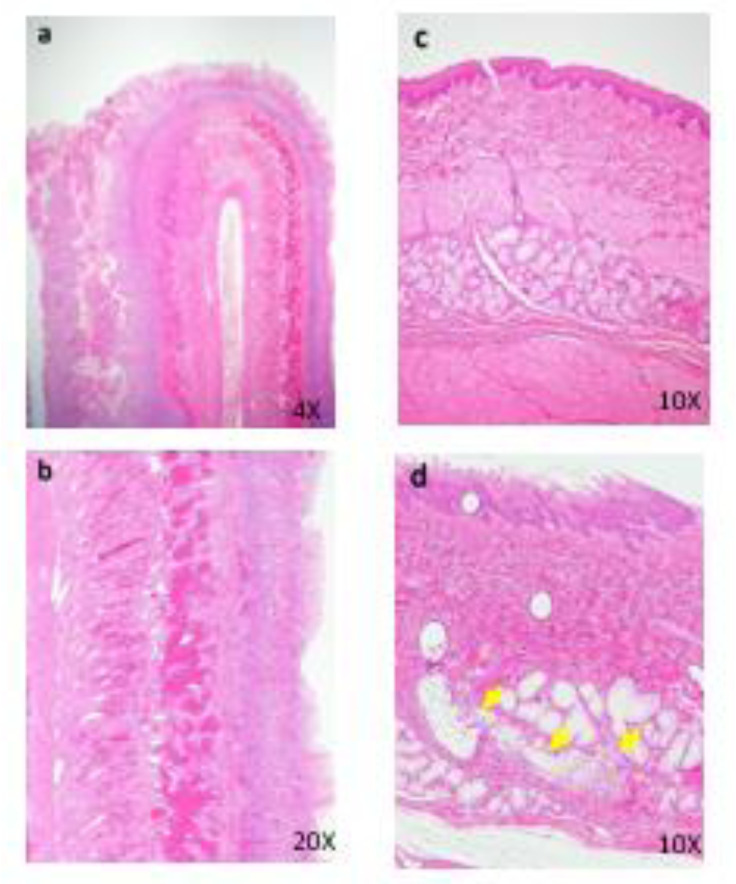
Histopathological findings in intestine. 50 mg/kg (a), Hyper view from lesion (b), a, The intussusception is seen. b, Peritonitis, necrosis and hemorrhage of intestinal walls are seen. Histopathological findings in esophagus. Control (c), 50 mg/kg (d), c, No abnormalities detected. d, Focal inflammation of esophageal gland (arrows) is seen.

No deaths occurred during the dosing period. Decreased body weight or suppressed body weight gain and decreased food consumption were observed in the 50 mg/kg group. For the organ weights and histopathology, decreased thymus weight and atrophy of the thymus were observed in the 50 mg/kg group. New histopathological findings that were not observed in the 4-week study, necrosis of the intestinal wall in ileum and focal inflammation of the esophageal gland in the esophagus was observed in the 50 mg/kg group (Figure 6(Fig. 6)). The plasma concentrations of OPC-163493 were elevated in a dose-related manner in both sexes, and no remarkable gender differences were observed. The NOAEL was estimated to be 10 mg/kg/day for males and 50 mg/kg/day for females. At the NOAEL, the plasma C_max_ and AUC_24h_ of OPC-163493 at Week 13 were respectively 7.604 µg/mL and 57.92 µg·h/mL in males and 13.97 µg/mL and 146.2 µg·h/mL in females. The results of observations, measurements, and examinations are shown in Supplementary Table 9.

### Single dose toxicity study in monkeys (Non-GLP)

No deaths occurred at any dose. Vomiting was observed at a low frequency for both sexes at 300 and 1000 mg/kg. Low food consumption was transiently observed in males at 1000 mg/kg. The results are shown in Supplementary Table 10.

### Preliminary 2-week repeated dose toxicity study in monkeys (Non-GLP)

**Figure 7:**
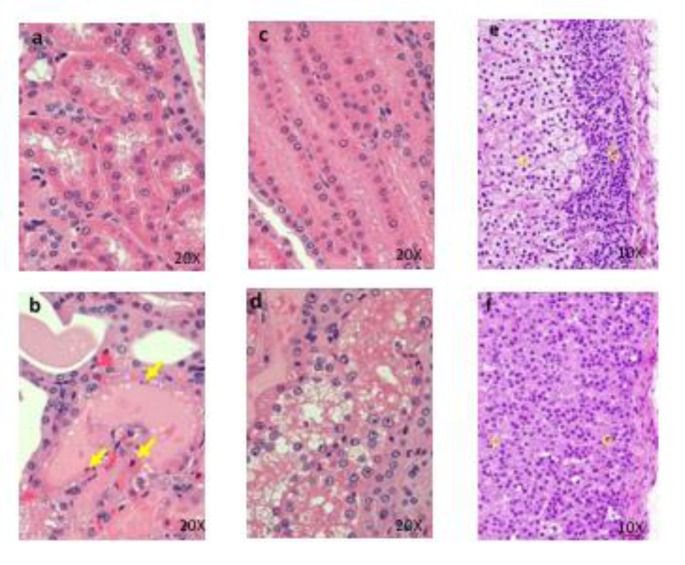
Histopathological findings of proximal convoluted tubules in kidney. Control (a), 1000 mg/kg (b) , a, No abnormalities detected. b, Degeneration and necrosis (arrows) of proximal convoluted tubular epithelium are seen. Histopathological findings of proximal straight tubules in kidney. Control (c), 1000 mg/kg (d), c, No abnormalities detected. d, Degeneration and necrosis of proximal convoluted tubular epithelium are seen. Histopathological findings in adrenal. control (e), 1000 mg/kg (f), e, No abnormalities detected, f, Hypertrophy of the cortical cells at glomerulosa are seen. g: zona glomerulosa, f: zona fasciculate

All males and females died or were sacrificed moribund at 100 and 1000 mg/kg/day. The following changes in these animals were observed in general condition, body weight, food consumption, hematology, blood biochemistry, necropsy, organ weights, and histopathology: vomiting, diarrhea, loose stool, twitch, a decrease in locomotor activity, prone position, lateral position, crouching position, decreased body weight, decreased food consumption, increased platelets, neutrophils, and monocytes, decreased lymphocytes, increased total bilirubin, BUN, CRE, TG, P, and K, decreased Na and Cl, distension of the stomach, emaciation, increased kidney weight, degeneration/necrosis of proximal convoluted tubular epithelium, degeneration/necrosis of proximal straight tubular epithelium, regeneration of proximal convoluted tubular epithelium, dilatation of distal tubule and hyaline cast in the kidney, and atrophy of mucosa in the esophagus and stomach (Figure 7(Fig. 7)). The results of observations, measurements, and examinations are shown in Supplementary Table 11.

### Four-week repeated dose toxicity study with 4-week recovery test in monkeys (GLP)

**Figure 8:**
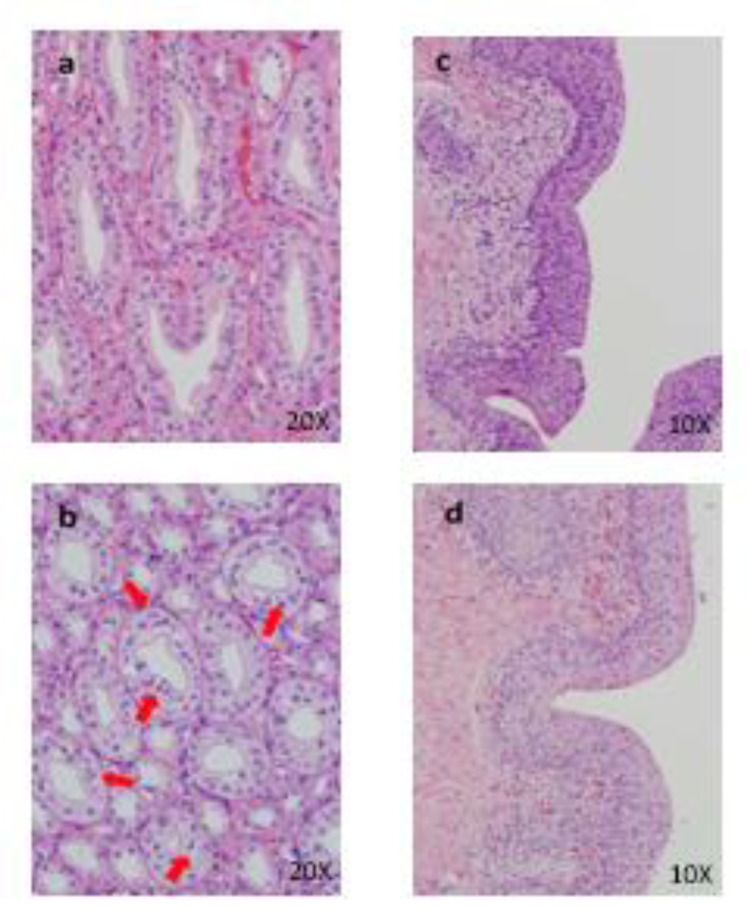
Histopathological findings in kidney. Control (a), 50 mg/kg (b), a, No abnormalities detected, b, Hyperplasia of mucosal epithelium are seen. Histopathological findings in urinary bladder. Control (c), 50 mg/kg (d), c, No abnormalities detected, d, Hyperplasia of mucosal epithelium are seen.

One male died at 50 mg/kg/day. Loose stool and/or diarrhea was observed in the 50 mg/kg group. New histopathological findings that were not observed in the 2-week study, degeneration of collecting tubular epithelium in the kidney and hyperplasia of mucosal epithelium in the urinary bladder were observed in the 50 mg/kg group (Figure 8(Fig. 8)). The changes noted during the dosing period were no longer evident during the recovery period in the clinical observations, food consumption, hematology, blood biochemistry, necropsy, or histopathology of the 50 mg/kg/day group. The plasma concentrations of OPC-163493 were elevated in a dose-related manner in both sexes, but no remarkable gender differences were observed. The NOAEL was estimated to be 30 mg/kg/day for both males and females. At the NOAEL, the plasma C_max_ and AUC_24h_ of OPC-163493 at Week 4 were respectively 12.07 µg/mL and 102.9 µg·h/mL in males and 8.223 µg/mL and 72.14 µg·h/mL in females. The results of observations, measurements, and examinations are shown in Table 4(Tab. 4).

### Thirteen-week repeated dose toxicity study in monkeys (GLP)

**Figure 9:**
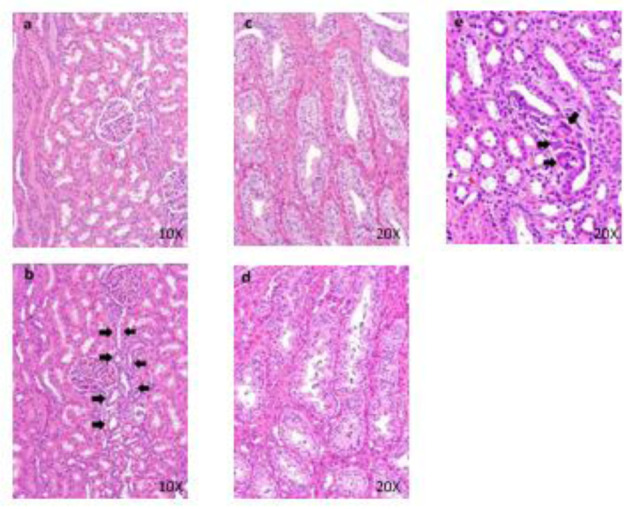
Histopathological findings in kidney. Control (a), 40 mg/kg (b), a, No abnormalities detected. b, Regeneration (arrows) of distal tubular epithelium are seen. Histopathological findings of collecting ducts in kidney. Control (c), 40 mg/kg (d), c, No abnormalities detected. d, Granular casts in collecting tubules are seen. Histopathological findings in cortex in kidney. 40 mg/kg (e), Foreign body granuloma (arrows) in the interstitium are seen.

No deaths occurred during the dosing period. The following changes in 1 male animal of the 40 mg/kg group were observed in the body weight, hematology, blood biochemistry, urinary biomarker, body temperature, organ weight, necropsy, and histopathology: decreased body weight, decreased RET, increased BUN, decreased CRE, Na, and Cl, increased calbindin, decreased body temperature, dilatation of distal tubules, regeneration of distal tubular epithelium, granular cast of collecting tubules, and foreign body granuloma of interstitium in the kidney (Figure 9(Fig. 9)). The plasma concentrations of OPC-163493 were elevated in a dose-related manner in both sexes, and no remarkable gender differences were observed. The NOAEL was estimated to be 20 mg/kg/day for males and 40 mg/kg/day for females. At the NOAEL, the plasma C_max _and AUC_24h_ of OPC-163493 at Week 13 were respectively 13.81 µg/mL and 91.44 µg·h/mL in males and 12.62 µg/mL and 96.10 µg·h/mL in females. The results of observations, measurements, and examinations are shown in Supplementary Table 12.

### Tissue concentrations of OPC-163493 and M29501 in liver and kidney (Non-GLP)

In the rats of the 300 mg/kg group, the liver concentrations of OPC-163493 and M29501 were 79.90 ± 38.23 and 51.43 ± 20.83 μg/g tissue for male, and 85.37 ± 27.88 and 72.94 ± 57.96 μg/g tissue for female, respectively. The kidney concentrations of OPC-163493 and M29501 were 30.50 ± 21.11 and 57.21 ±21.08 μg/g tissue for male, and 27.62 ± 10.53 and 66.21 ± 79.10 µg/g tissue for female, respectively. In the dogs of the 100 mg/kg group, the liver concentrations of OPC-163493 and M29501 were 3.598 ± 0.074 and 4.550 ± 2.475 μg/g tissue for male, and 7.197 ± 6.227 and 5.626 ± 6.698 μg/g tissue for female, respectively. The kidney concentrations of OPC-163493 and M29501 were 2.411 ± 1.146 and 1.774 ± 1.586 μg/g tissue for male, and 4.408 ± 5.535 and 0.7950 ± 1.0896 μg/g tissue for female, respectively. In the monkeys of the 50 mg/kg group, the liver concentrations of OPC-163493 and M29501 were 6.485 ± 0.656 and 112.2 ± 51.4 μg/g tissue for male, and 6.721 ± 1.916 and 66.68 ± 41.48 μg/g tissue for female, respectively. The kidney concentrations of OPC-163493 and M29501 were 0.3927 ± 0.0661 and 50.23 ± 34.07 μg/g tissue for male, and 0.7075 ± 0.5396 and 21.61± 10.58 μg/g tissue for female, respectively. The results ofmeasurements are shown in Table 5(Tab. 5).

## Discussion

In the pharmacokinetics and pharmacodynamics studies, the OPC-163493 plasma concentration of 4.54 µg·h/mL (AUC_24h_) affected respiratory metabolism in ZDF rats (Kanemoto et al., 2019[12]; Okamoto et al., 2021[16]). In rats, dogs, and monkeys, safety ratios between the effective dose and NOAEL were 100:1, 13:1, and 20:1, respectively, in terms of total exposure (AUC_24h_) (Supplementary Figure 1). The highest exposure levels of OPC-163493 caused toxicities in animals. These toxicities could be monitored in clinical trials and there is a sufficiently large safety margin between the effective dose and the toxic dose (Supplementary Figure 2). In the single dose toxicity study in rats, rigor mortis was observed in the 1000 and 2000 mg/kg groups immediately after they died. It has been reported that mUncouplers produced immediate rigor mortis after the animal had died (Parker, 1965[18]). Respiratory failure was considered as one possible cause of death, since labored breathing, partial lung collapse, and dark red foci in the lung were observed. In addition, mUncouplers are known to cause an acute failure of muscular activity leading to death (Parker et al., 1951[19]). Therefore, an acute failure of muscular activity was considered as another possible cause of death since rigor mortis was observed immediately after the animal died.

In the repeated dose toxicity study in rats, increased body temperatures in the 300 mg/kg group were observed at 4 hours after dosing in Week 2 and Week 4. The pharmacological efficacy of OPC-163493 is related to its effects on cellular energy metabolism (Kanemoto et al., 2019[12]; Okamoto et al., 2021[16]). It is known that pyrexia was induced in rats by subcutaneous injection of 2,4-dinitrophenol (Tainter and Cutting, 1933[25]). Therefore, this change was considered to be an excessive pharmacological effect of the test article. Increased Hb, Ht, and/or RBC were observed in the 100 and 300 mg/kg groups. These changes were considered to be an adaptation to the oxygen demand, based on the fact that OPC-163493 induced hyperventilation in the 300 mg/kg group in the safety pharmacological study and an increase in the respiratory rate was induced in rats by the administration of 2,4-dinitrophenol (Kaiser, 1964[11]). Increased blood chemistry findings were positively associated with focal necrosis of the hepatocytes in the 300 mg/kg group. In addition, hypertrophy of the hepatocytes was observed in the 100 and 300 mg/kg groups. Increased liver weight was also observed in the 300 mg/kg group. It was unclear whether these changes were of any toxicological significance since electron microscopy revealed no abnormalities in the organelles of the hypertrophied hepatocytes. Fatty change of the hepatocytes in the midzone of the lobules was also observed in the 300 mg/kg group. An increase in lipid droplets in the hepatocytes of the intermediate zone in the liver was observed in the electron microscopic examination of the fatty change of the hepatocytes. Granular eosinophilic cytoplasm of the hepatocytes was observed in the 300 mg/kg group. Enlargement and an increase in the number of mitochondria in the hepatocytes of the central zone in the liver were observed in the electron microscopic examination of the granular eosinophilic cytoplasm of the hepatocytes. Therefore, the granular eosinophilic cytoplasm of the hepatocytes was shown to be caused by the enlargement and increased number of mitochondria. Swelling of mitochondria in the hepatocytes of the central zone in the liver was observed in the electron microscopic examination of the hepatocytes. It is known that the swelling of mitochondria with disruption of cristae and irregular densities in the matrix in the hepatocytes can be caused by an mUncoupler (Maronpot et al., 1999[15]; Cattley and Popp, 2002[1]). Degeneration of the lens fiber was observed in the 300 mg/kg group. It is known that cataracts were induced in ducks and rabbits by the administration of 2,4-dinitrophenol (Gehring and Buerge, 1969[5]). In addition, apoptosis in the ganglion cell layer and inner nuclear layer of the retina was observed in the 300 mg/kg group. Necrosis of the islet cells in the pancreas was observed in the 300 mg/kg group, but the Glu concentrations showed no changes. The necrosis of the islet cells was reported to be caused by oxidative stress, endoplasmic reticulum stress (ER), β cell exhaustion, and/or β cell hyperactivity (Szkudelski, 2001[24]; Papa, 2012[17]). Hyperplasia of the transitional cells in the renal pelvis and urinary bladder were observed in the 300 mg/kg group. The change was also observed in the preliminary 2-week repeated oral dose toxicity study. These changes were reported to result from the irritation caused by drug crystallization or uroliths (Greaves, 2012[6]). Atrophic change in the ovaries was observed in the 300 mg/kg group. The change was considered to be a secondary effect caused by the suppressed body weight gain and the atrophic changes in the thymus and/or hypertrophy of the zona fasciculata/reticularis in the adrenals. Degeneration/necrosis of the muscle fibers was observed in the 300 mg/kg group. The change was considered to be generated at the agonal stage since it was not accompanied by any vital responses such as infiltration of the inflammatory cells. Therefore, the change was considered to be related to muscular rigidity.

In the single dose toxicity study in dogs, vomiting, diarrhea, loose stool, and low food consumption were transiently observed in the 300 and 600 mg/kg groups.

In the repeated dose toxicity study in dogs, vomiting, mucous stool, diarrhea, and loose stool were observed in the 100 mg/kg groups. Decreased body weight and decreased food consumption were observed in the 100 mg/kg groups. Of the animals given 100 mg/kg, 1 male had diarrhea every day during Week 4 and emaciation and hypoactivity were also observed. This male given 100 mg/kg also had necrotizing arteritis in multiple organs including the heart, aorta, esophagus, stomach, kidney, prostate, urinary bladder, and skeletal muscle as well as meningitis with hemorrhage in the brain. Furthermore, increases of neutrophils in the sinusoid and microgranuloma in the liver, granulocytic hyperplasia in the bone marrow, and increases of neutrophils in the red pulp of the spleen were observed in this male, and these changes as well as increased body temperature at Week 4 were considered to be consequences of the systemic inflammatory state caused by the necrotizing arteritis in multiple organs. In the preliminary 2-week dosing study, meningitis with hemorrhage in the brain and meningitis in the spinal cord were also observed at dosages of 100 and/or 600 mg/kg. The findings of necrotizing arteritis in multiple organs with inflammatory changes (neutrophilia, increased α2, 3-G fraction and meningitis) observed in this study were similar to the findings previously reported in cases of idiopathic canine polyarteritis (ICP) (Hayes et al., 1989[7]; Tipold and Schatzberg, 2010[27]; Son, 2004[23]; Kemi et al., 1990[13]). The right atrial and right ventricular myocardial necrosis and right and left ventricular myocardial fibrosis in the heart were observed in 1 male given 100 mg/kg. This animal was also emaciated. It was considered that these myocardial changes were caused by ischemia since the necrotizing arteritis in the coronary artery was evident in this male. Necrosis of the mucosal epithelium in the renal pelvis was observed in 1 female given 100 mg/kg. A similar change in the urinary tract was also observed in the preliminary 2-week toxicity study of OPC-163493 in beagle dogs at dosages of 100 and 600 mg/kg. It was anticipated that the excreted urine or urinary ingredients affected the mucosal epithelium in the renal pelvis, and it was also reported that a similar change was the result of irritation caused by drug crystallization or uroliths (Greaves, 2012[6]). 

In the single dose toxicity study in monkeys, vomiting was observed in both sexes at all dose levels of the test article on the day of administration and/or the following day.

In the repeated dose toxicity study in monkeys, decreased food consumption, decreased locomotor activity, and moribundity were observed in 1 male at 50 mg/kg. In the animal that died, treatment-related abnormalities were noted in the kidneys, urinary bladder, ureter, adrenals, lymphoid organs, pancreas, and submandibular and parotid glands. The changes considered attributable to the direct effect of the test article or urine metabolite included degeneration of proximal convoluted tubules or straight tubular epithelium, regeneration of proximal tubular epithelium, dilatation of distal tubules, degeneration/hyperplasia of the papilla urothelium, and degeneration of collecting tubular epithelium in the kidney, and degeneration/hyperplasia of mucosal epithelium in the urinary bladder, and degeneration of mucosal epithelium in the ureter. Copious amounts of fluid and electrolytes are reabsorbed by the proximal convoluted tubules in the kidney. The degeneration of the proximal convoluted tubules or straight tubular epithelium found in this study suggest that the changes in the kidney are associated with the blood chemistry and hematology findings. In the animal found dead, degeneration of the proximal convoluted tubules or straight tubular epithelium in the kidney was noted in the histopathological examination, and decreased serum sodium and increased potassium were noted in the blood chemistry on the day of death. Thus, it was suggested that the renin-angiotensin system in the kidney had been stimulated, which induced aldosterone hypersecretion from the adrenal cortical cells. These changes were also observed in the preliminary 2-week repeated oral dose toxicity study in monkeys.

The target organs of OPC-163493 were liver, blood vessels, and kidney in rats, dogs, and monkeys, respectively. As a part of the investigation of the mechanism of toxicity, the tissue concentrations of OPC-163493 in liver and kidney were measured in rats, dogs, and monkeys. The concentration of OPC-163493 in the liver of rats was 10 times higher than that of dogs and monkeys, suggesting that OPC-163493 induced hepatotoxicity. On the other hand, the concentration of OPC-163493 in the kidneys of monkeys was more than 10-fold lower than that in rats and dogs. The tissue concentrations of M29501 in liver and kidney were measured in rats, dogs, and monkeys. The concentration of M29501 in the kidneys of monkeys was almost the same as those in rats. Therefore, the mechanism of nephrotoxicity in monkeys remains unknown. The sufficient safety margin and monitorable toxicity and recovery suggest that clinical development in humans can proceed safely. We hope that new toxicity findings for OPC-163493 as an mUncoupler agent will lead to these agents being considered as a new therapeutic option in the future.

## Declaration

### Acknowledgments

The authors are grateful to Toshiki Sudo, Eiji Kashiyama and Ken Umehara for recommending the submission of this article.

### Funding

Not applicable.

### Author contributions

YI designed the experiments and wrote the manuscript. JK and NI designed the experiments, collected and analyzed the data. MS, SH, HY and TS collected and analyzed the data. SS synthesized OPC-163493. TO and NK revised the manuscript.

### Availability of data and material 

Data and material that support the findings of the study are available from the corresponding author upon reasonable request.

### Competing interests 

All authors are employees of Otsuka Pharmaceutical Co., Ltd.

### Ethics approval 

Rats' and dogs' studies were conducted in compliance with the guidelines for animal care and use in Otsuka Pharmaceutical Co, Ltd, and was approved by the institutional animal care and use committee of the testing facility. Monkeys studies were conducted in compliance with the guidelines for animal care and use in LSI Medience Co, and was approved by the institutional animal care and use committee of the testing facility. In addition, all animal experimental procedures were carried out in GLP facilities. 

## Supplementary Material

Supplementary tables

Supplementary figures

## Figures and Tables

**Table 1:**
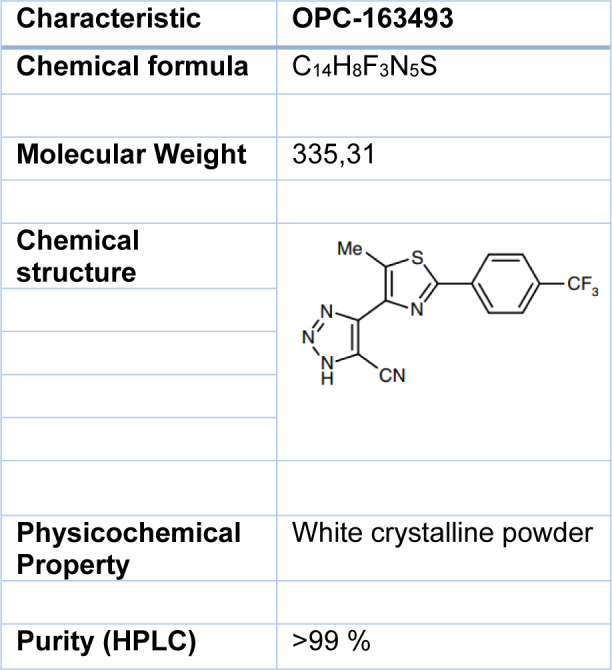
Physicochemical property of OPC-163493

**Table 2:**
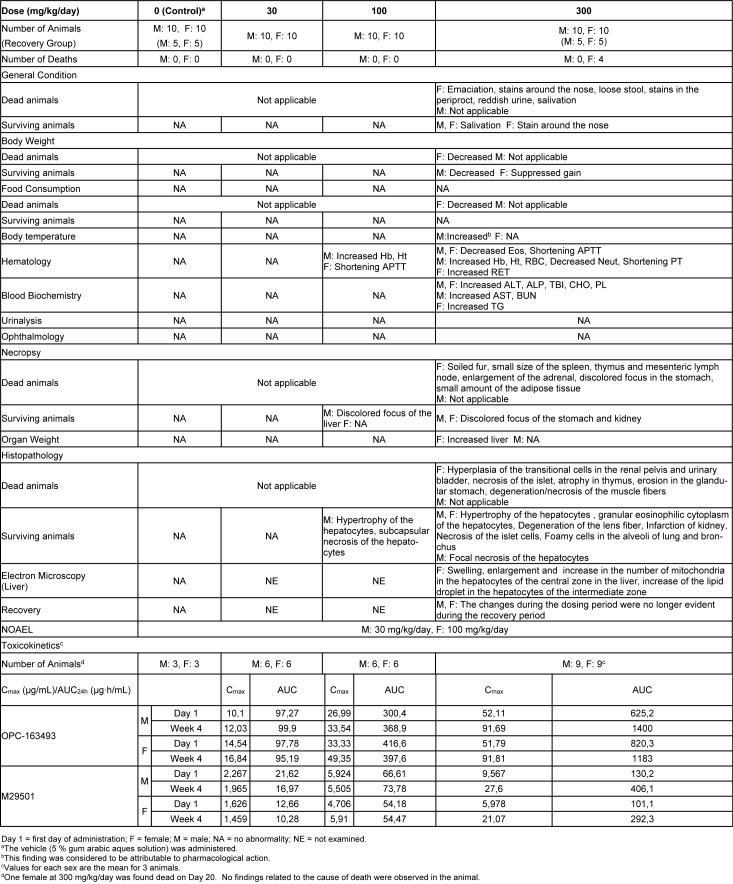
Four-week repeated-dose oral toxicity study in rats with 4-week recovery test

**Table 3:**
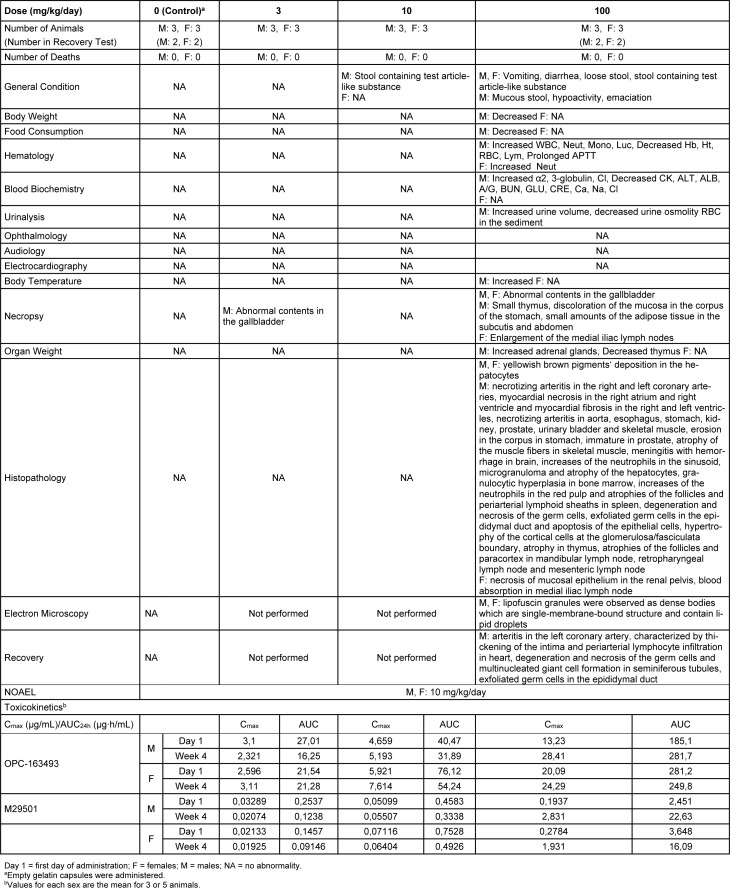
Four-week repeated-dose oral toxicity study in dogs with 4-week recovery test

**Table 4:**
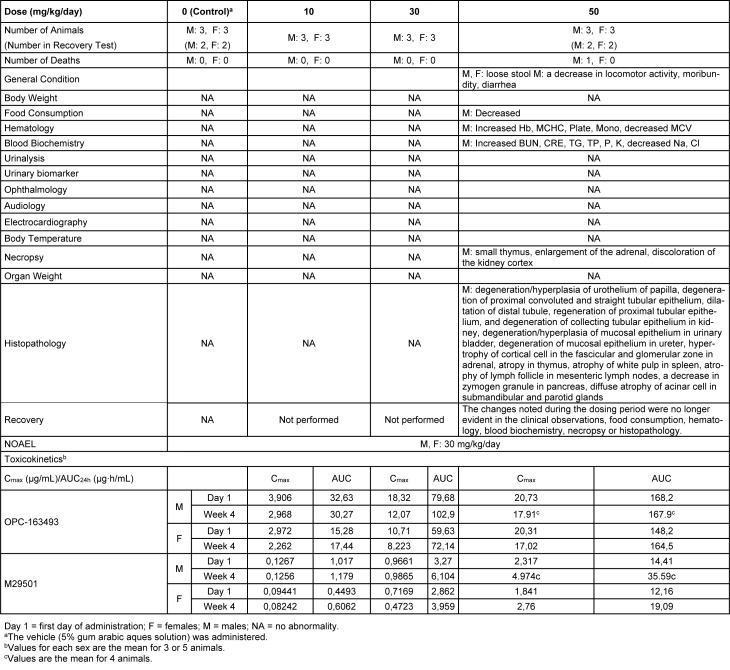
Four-week repeated-dose oral toxicity study in monkeys with 4-week recovery test

**Table 5:**
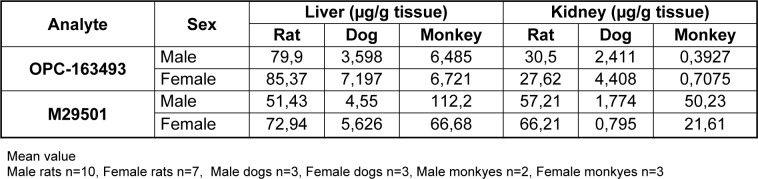
The average concentrations of OPC-163493 and M29501 in the tissues
