# Supplementary material for: Preclinical safety profile of a liver-localized mitochondrial uncoupler: OPC-163493
Source: EXCLI J. 2022 Jan 11;21:213–35. doi: 10.17179/excli2021-4414 (PMC8859645; doi:10.17179/excli2021-4414)

EXCLI Journal 2022;21:213-235 – ISSN 1611-2156
Received: October 15, 2021, accepted: December 23, 2021, published: January 11, 2022

Supplementary information to:

Original article:

PRECLINICAL SAFETY PROFILE OF A LIVER-LOCALIZED MITOCHONDRIAL UNCOUPLER: OPC-163493

Yuki Inoue[1,*], Junichi Kino[2], Nobuya Ishiharada[3], Makoto Sato[1], Suguru Hatanaka[4], Hiroyuki Yokoi[4], Takahiro Shimada[4], Seiji Sato[5], Takashi Okamoto[6], Naohide Kanemoto[6]

1 Department of Drug Safety Research, Nonclinical Research Center, Tokushima Research Institute, Otsuka Pharmaceutical Co., Ltd., Tokushima, Japan
2 Product Strategy Team 1, Product Strategy & Intelligence Office, Regulatory Affairs Department, Otsuka Pharmaceutical Co., Ltd., Tokyo, Japan
3 Department of Investigative Toxicology, Nonclinical Research Center, Tokushima Research Institute, Otsuka Pharmaceutical Co., Ltd., Tokushima, Japan
4 Department of Drug Metabolism and Pharmacokinetics, Nonclinical Research Center, Tokushima Research Institute, Otsuka Pharmaceutical Co., Ltd., Tokushima, Japan
5 Medicinal Chemistry Research Laboratories, New Drug Research Division, Otsuka Pharmaceutical Co., Ltd., Tokushima, Japan
6 Department of Lead Discovery Research, New Drug Research Division, Otsuka Pharmaceutical Co., Ltd., Tokushima, Japan

* **Corresponding author:** Yuki Inoue, Department of Drug Safety Research, Nonclinical Research Center, Tokushima Research Institute, Otsuka Pharmaceutical Co., Ltd., 463-10 Kagasuno Kawauchi-cho, Tokushima, 771-0192, Japan.
E-mail: Inoue.Yuki@otsuka.jp

https://dx.doi.org/10.17179/excli2020-4414

This is an Open Access article distributed under the terms of the Creative Commons Attribution License (http://creativecommons.org/licenses/by/4.0/).

EXCLI Journal 2022;21:213-235 – ISSN 1611-2156
Received: October 15, 2021, accepted: December 23, 2021, published: January 11, 2022

Supplementary Figure 1: Safety margins between efficacy dose and NOAEL based on plasma AUC µg·h/mL

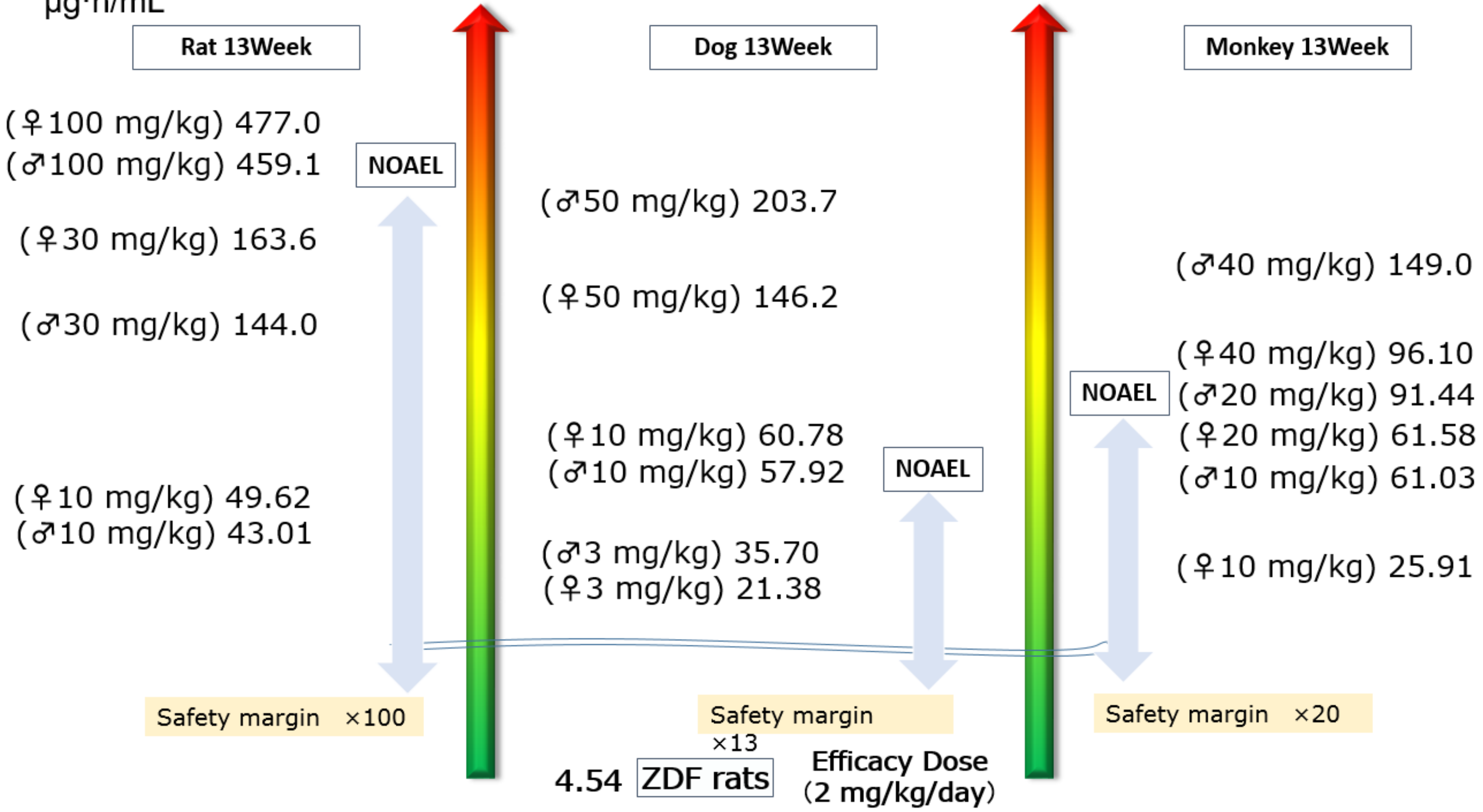

EXCLI Journal 2022;21:213-235 – ISSN 1611-2156
Received: October 15, 2021, accepted: December 23, 2021, published: January 11, 2022

Supplementary Figure 2: Safety margins between efficacy dose and toxicity dose based on plasma AUC µg·h/mL

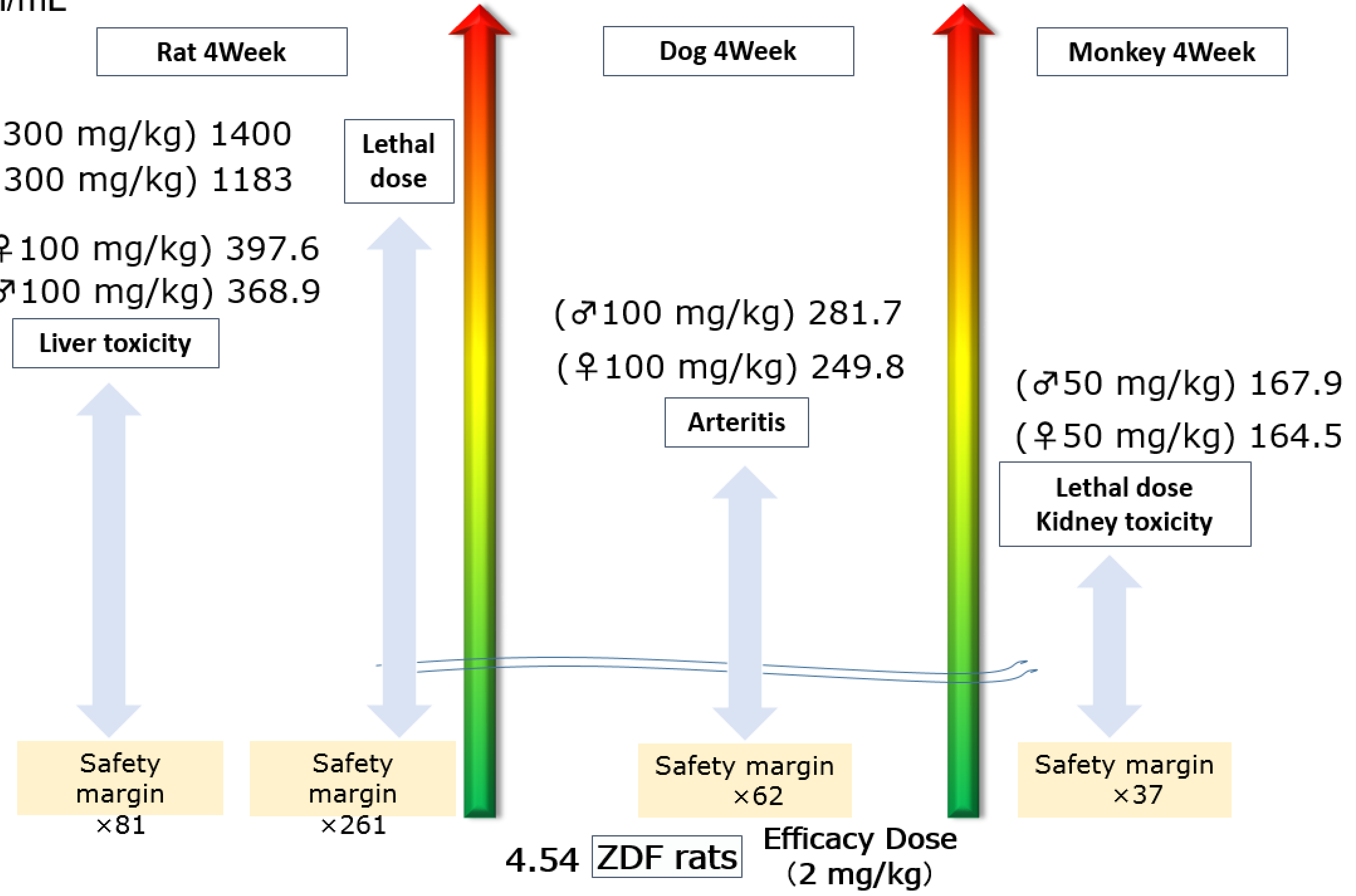

Supplement: Supplementary figures [file EXCLI-21-213-s-002.pdf]
